# Targeted deletions of complement lectin pathway genes improve outcome in traumatic brain injury, with MASP-2 playing a major role

**DOI:** 10.1186/s40478-020-01041-1

**Published:** 2020-10-28

**Authors:** D. Mercurio, M. Oggioni, S. Fumagalli, N. J. Lynch, S. Roscher, D. Minuta, C. Perego, S. Ippati, R. Wallis, W. J. Schwaeble, M.-G. De Simoni

**Affiliations:** 1 Department of Neuroscience, Istituto di Ricerche Farmacologiche Mario Negri IRCCS, via Mario Negri 2, 20156 Milan, Italy; 2 Department of Respiratory Sciences, University of Leicester, University Road, LE1 9HN Leicester, UK; 3 Department of Veterinary Medicine, University of Cambridge, Madingley Road, CB3 0ES Cambridge, UK; 4 Present Address: San Raffaele Scientific Institute, San Raffaele Hospital, 20132 Milan, Italy; 5 Present Address: National Research Council (CNR), Institute of Neuroscience, 20129 Milan, Italy

**Keywords:** Traumatic brain injury, Neuroinflammation, Lectin pathway, Complement cascade, MBL-associated serine protease, Neurological deficits, Pharmacological target

## Abstract

The lectin pathway (LP) of complement activation is believed to contribute to brain inflammation. The study aims to identify the key components of the LP contributing to TBI outcome as possible novel pharmacological targets. We compared the long-term neurological deficits and neuropathology of wild-type mice (WT) to that of mice carrying gene deletions of key LP components after experimental TBI. WT or MASP-2 (Masp2^−/−^), ficolin-A (Fcna^−/−^), CL-11 (Colec11^−/−^), MASP-1/3 (Masp1^−/−^), MBL-C (Mbl2^−/−^), MBL-A (Mbl1^−/−^) or MBL^−/−^ (Mbl1^−/−^/Mbl2^−/−^) deficient male C57BL/6J mice were used. Mice underwent sham surgery or TBI by controlled cortical impact. The sensorimotor response was evaluated by neuroscore and beam walk tests weekly for 4 weeks. To obtain a comparative analysis of the functional outcome each transgenic line was rated according to a health score calculated on sensorimotor performance. For selected genotypes, brains were harvested 6 weeks after injury for histopathological analysis. MASP-2^−/−^, MBL^−/−^ and FCN-A^−/−^ mice had better outcome scores compared to WT. Of these, MASP-2^−/−^ mice had the best recovery after TBI, showing reduced sensorimotor deficits (by 33% at 3 weeks and by 36% at 4 weeks). They also showed higher neuronal density in the lesioned cortex with a 31.5% increase compared to WT. Measurement of LP functional activity in plasma from MASP-2^−/−^ mice revealed the absence of LP functional activity using a C4b deposition assay. The LP critically contributes to the post-traumatic inflammatory pathology following TBI with the highest degree of protection achieved through the absence of the LP key enzyme MASP-2, underlining a therapeutic utility of MASP-2 targeting in TBI.

## Introduction

Traumatic brain injury (TBI) is associated with a primary biomechanical injury that can involve contusion and laceration, diffuse axonal injury, brain swelling and intracranial haemorrhage [1–3] followed by a secondary injury, which is caused by the activation of several molecular and cellular cascades contributing to brain damage and its development over time [4–6]. Secondary insult response typically includes blood–brain barrier (BBB) breakdown, oxidative stress, glutamate excitotoxicity, and neuroinflammation [7–9]. Since the secondary damage evolves days after the impact, there are windows of opportunity for pharmacological therapeutic intervention.

The complement system, an important component of the innate and adaptive immune response, is a major coordinator of post-traumatic neuroinflammation and secondary neuropathology after TBI [10–14]. Even in absence of infection (which can be an additional complication following TBI) the complement system can be activated by endogenous danger signals called damage-associated molecular patterns (DAMPs) [15]. Depending on the signals, complement activation may occur through three different pathways, the classical, the alternative and the lectin pathway (LP), each composed of specific initiators and effector enzymes. Carbohydrate structures or acetylated proteins exposed on the surface of damaged cells, including apoptotic or necrotic cells and stressed endothelium [16], are typical DAMPs recognized by the LP. Recognition of these signals by initiator molecules leads to activation of the associated serine proteases MASPs (MBL-associated serine proteases) and subsequent activation of the complement cascade.

There is a growing body of evidence showing that LP activation critically contributes to disease severity in acute brain injury. Genetic deletion of LP subcomponents (MBL or MASP-2) [17–19] or their pharmacological targeting [17, 18] are protective in experimental models of brain ischemia. In addition to its role as an LP initiator, the recognition subcomponent MBL was shown to possess direct activity, driving platelet-dependent inflammation and vascular damage following ischemic injury [16, 20]. Of note, TBI shares blood perfusion deficits and metabolic derangements with ischemic injury [21], thus suggesting that similar mechanisms might be involved in the traumatic pericore tissue [22], an area subjected to post-injury hypoxia [23].

In human TBI contusions, the LP recognition molecules (MBL, ficolin-1, ficolin-2, ficolin-3 and CL-11/CL-10) and the enzymes MASP-2 and MASP-3 have been found deposited inside and outside the cerebral vessels. Importantly, MBL, ficolin-2 and ficolin-3 levels are elevated in the brains of TBI compared to non-TBI patients and perivascular MASP-2 deposition increase with injury severity [23]. These results demonstrate the involvement of the LP in TBI pathology, although the specific recognition molecules or enzymes which most prominently contribute to the TBI *sequelae* remain elusive. The comparative analysis shown here, identifies novel targets for therapeutic intervention, aiming to reduce post traumatic inflammatory responses, brain tissue loss, and to ameliorate loss of cognitive function following TBI. In this study, we compared the neurobehavioral outcome and health score up to 4 weeks after TBI of wild-type (WT) mice and of mice knocked-out for MASP-2 (*Masp2*^−/−^), ficolin-A (*Fcna*^−/−^), CL-11 (*Colec11*^−/−^), MASP-1/3 (*Masp1*^−/−^), MBL-C (*Mbl2*^−/−^), MBL-A (*Mbl1*^−/−^) or MBL^−/−^ (*Mbl1*^−/−^/*Mbl2*^−/−^). Our results demonstrate that MASP-2 deficiency brings the highest protective phenotype, countering long-term neuroinflammatory injury following TBI, as shown by reduced neuronal deficits and neuronal cell loss compared to wild-type mice. MBL^−/−^ and FCN-A^−/−^ mice were also significantly protected and presented good outcome scores, indicating that these molecules might be relevant initiators of the inflammatory response to TBI.

## Methods

### Mice

Procedures involving animals and their care were conducted in conformity with institutional guidelines in compliance with national and international laws and policies (prot.9F5F5.81 authorisation n° 753/2017-PR). A total of 61 WT and 57 KO mice were studied. We used male 9 week old C57BL/6J mice weighing 22–28 g, either WT (purchased from Charles Rivers-Italy) or with targeted deletion of MASP-2, ficolin-A, CL-11, MASP-1/3 (*Masp2*^−/−^, *Fcna*^−/−^, *Colec11*^−/−^, *Masp1*^−/−^ Biomedical Services, University of Leicester) MBL-C and MBL-A, (*Mbl2*^−/−^ and *Mbl1*^−/−^ obtained at Mario Negri Institute by crossing MBL^−/−^ mice with WT mice and selecting appropriate colony founders). The protocols and details of this report are in accordance with ARRIVE guidelines (http://www.nc3rs.org.uk/page.asp?id=1357, check list provided as supplementary file).

### Experimental traumatic brain injury

Mice were anesthetized with isoflurane inhalation (induction 5%; maintenance 2%) in an N_2_O/O_2_ (70/30%) mixture and placed in a stereotactic frame. Mice were then subjected to craniotomy followed by induction of controlled cortical impact (CCI) brain injury as previously described [24–27]. Briefly, the injury was induced using a 3 mm diameter rigid impactor driven by a pneumatic piston rigidly mounted at an angle of 20° from the vertical plane and applied vertically to the exposed dura mater, between bregma and lambda, over the left parieto-temporal cortex. We set an impactor velocity of 5 m/s and deformation depth 1 mm, resulting in a severe level of injury [28, 29]. The craniotomy was then covered with a cranioplasty and the scalp sutured. Sham-operated mice received identical anesthesia and surgery without craniotomy and brain injury.

### Behavioural tests

Sensorimotor deficits were assessed by neuroscore and beam walk tests as described previously [22, 28, 30] *Neuroscore* Mice were scored from 4 (normal) to 0 (severely impaired) for each of the following: (1) forelimb function during walking on the grid and flexion response during suspension by the tail; (2) hindlimb function during walking on the grid and extension during suspension by the tail; (3) resistance to lateral right and left push. The best score is 12.

*Beam walk* The test measures the number of foot faults of the mouse walking twice on an elevated, narrow wooden beam (5 mm wide and 100 cm long). Before each test, mice are trained in three habituation trials. Data are expressed as the sum of the number of foot faults during the two tests. The best score is 0.

### Tissue processing

At 6 weeks after surgery, under deep anesthesia (Ketamine 20 mg + Medetomidine 0.2 mg), animals were transcardially perfused with 30 mL of phosphate buffer saline (PBS) 0.1 mol/L, pH 7.4, followed by 60 mL of chilled paraformaldehyde (4%) in PBS. The brains were carefully removed from the skull and post-fixed for 6 h at 4 °C, then transferred to 30% sucrose in 0.1 mol/L phosphate buffer for 24 h until equilibration. The brains were frozen by immersion in isopentane at −45 °C for 3 min before being sealed into vials and stored at −80 °C until use. Coronal brain 20 μm-thick cryosections were cut serially (from bregma + 1.2 mm to bregma − 4 mm) at 200 μm intervals and stained with Cresyl violet (Sigma-Aldrich) using standard histological protocols [26, 31].

### Contusion volume

Eight coronal section from bregma + 0.6 to − 4.0 mm were acquired with an Olympus BX-61 Virtual Stage microscope using a 2 × objective lens, with a pixel size of 3.49 µm. Contusion volume was analysed as previously described [22].

### Neuronal count

The neuronal cell count was performed at 6 weeks after TBI. Three 20 μm-thick coronal sections at 0.4, 1.6, and 2.8 mm posterior to bregma and stained with Cresyl violet (Sigma-Aldrich, St. Louis, MO) were selected from each mouse brain to quantify neuronal cell loss. The entire sections were acquired with an Olympus BX-61 Virtual Stage microscope using a 20 × objective lens, with a pixel size of 0.346 μm. Acquisition was done over 10 μm thick stacks, with a step size of 2 μm. The different focal planes were merged into a single stack by mean intensity projection to ensure consistent focus throughout the sample. Neuronal count was performed by segmenting the cells over a cortical region proximal to the lesion and in the corresponding contralateral hemisphere and excluding the round-shaped signal sized below the area threshold of 25 mm^2^ that is known to be associated with glial cells as reported previously [32]. Quantification was performed by Fiji software. Data were expressed as the total number of neurons quantified in the selected cortical region.

### Microglia and astrocyte immunohistochemical analysis

Immunohistochemistry was performed on 20 μm-thick coronal sections from perfused mouse brains. The sections were incubated overnight at 4 °C with primary monoclonal antibody anti-mouse glial fibrillary acid protein (GFAP, 0.5 µg/ml, Millipore, Billerica, MA, USA) or anti-mouse CD11b (1.25 µg/ml, Bio rad, Hercules, CA, USA). Biotinylated secondary antibodies (7.5 µg/ml, Vector Laboratories) were used. GFAP and CD11b immunopositive cells were identified by reaction with 3,3 diaminobenzidine-tetrahydrochloride (DAB, Vector Laboratories, Burlingame, CA, USA) as previously described [33]. Negative control studies, without the primary antibody, were performed in parallel. Three 20 μm-thick coronal sections at 0.4, 1.6, and 2.8 mm posterior to bregma were selected from each mouse brain for GFAP and CD11b quantification. The entire sections were acquired with an Olympus BX-61 Virtual Stage microscope using a 20 × objective lens, with a pixel size of 0.346 μm. Acquisition was done over 10 μm thick stacks, with a step size of 2 μm. The different focal planes were merged into a single stack by mean intensity projection to ensure consistent focus throughout the sample. The ipsilateral cortex was analyzed over an area included within a 350 µm radius from the contusion edge. Images were analyzed using Fiji software. GFAP and CD11b immunostained area were expressed as positive pixels/total assessed pixels and reported as the percentage of total stained area [28]. Microglia shape descriptor analysis was performed on image processed through the algorithm previously described [21]. Once segmented, the cells were measured for the following parameters: area, perimeter, circularity, Feret’s diameter (max caliper), aspect ratio and solidity. Mean single-cell values for each parameter were used for statistics.

### MBL-C deposition in the brain

The brain coronal sections were incubated overnight at 4 °C with primary monoclonal antibody anti-mouse MBL-C (1 µg/ml; Hycult Biotechnology, Uden, The Netherlands) followed by a secondary biotinylated antibody against rat IgG. Positive cells were stained with Tyramide Cyanine 5 (Cy5, 1:300, Perkin Elmer, Milan, Italy). Cell nuclei were stained with 40-6-diamidino-2-phenylindole (Hoechst, 1 mg/ml, Invitrogen, Carlsbad, CA). For negative control staining, the primary antibody was omitted, and no staining was observed. Three 20 μm-thick coronal sections at 0.4, 1.6, and 2.8 mm posterior to bregma were selected from each mouse brain for MBL-C quantification. Confocal microscopy was done with a Nikon A1 confocal scan unit with a 20 × 0.5 numerical aperture (NA) objective, managed by NIS elements software. Tissues were imaged at laser excitation of 405 (for nuclei) and 647 (for MBL-C) [34]. Image acquisition was done at 12-bit, keeping the fluorescent signal in a non-saturated range (0–1000 greyscale values). The acquisition was done over an area sized 2 × 2.5 mm, positioned in the ipsilateral hemisphere along the cortical region proximal to the lesion, with a pixel size of 0.62 μm. Acquisition was done over 8.3 μm thick stacks, with a step size of 2.075 μm. The different focal planes were merged into a single stack by maximum intensity projection to ensure consistent focus throughout the sample. Immunostaining for MBL-C was quantified by assessing fluorescence intensity using Fiji software. To subtract the background signal, a minimum threshold was applied based in the highest grayscale value of background [28]. MBL-C signal was reported as fluorescence integrated density.

### Lectin pathway activity assay

Lectin Pathway activation was quantified using the C4 cleavage assay developed by Petersen et al. [35]. The assay measures the ability of MASP-2 to cleave human C4 added in replacement of endogenous C4 which is inactivated. This procedure provides a clean measure of MASP-2 activity without interference from endogenous C4. Nunc MaxiSorb microtiter plates were coated with 100 µl of mannan in coating buffer. After overnight incubation, wells were blocked with 0.1% human serum albumin (HSA) in TBS (10 mM Tris–Cl, 140 mM NaCl, pH 7.4), then washed with TBS containing 0.05% Tween 20 and 5 mM CaCl_2_ (wash buffer). Plasma samples were diluted in 20 mM Tris–Cl, 1 M NaCl, 10 mM CaCl_2_, 0.05% Triton X-100, 0.1% HSA, pH 7.4, which prevents activation of endogenous C4. The diluted samples were added to the plate and incubated overnight at 4 °C. The next day, the plates were washed thoroughly with wash buffer, then 0.1 µg of purified human C4 [36] in 100 µl of 4 mM barbital, 145 mM NaCl, 2 mM CaCl_2_, 1 mM MgCl_2_, pH 7.4, was added to each well. After 1.5 h at 37 °C, the plates were washed again, and C4b deposition was detected using alkaline phosphatase-conjugated chicken anti-human C4c (Immunsystem, Uppsala, Sweden) and the colorimetric substrate pNPP.

### Health score

To obtain a comparative analysis of the functional outcome, each transgenic line was rated according to a health score calculated on sensorimotor performance, as shown previously [19]. The neuroscore and beam walk performance data sets obtained in WT mice (total number = 61) were stratified into four groups according to quartiles. Each quartile was attributed a score ranging from 4 to 1 corresponding to the best to the worst outcome, respectively. Each mouse (including WT and KO) obtains a final score which is the sum of the weighted scores of the two parameters, e.g. the neuroscore accounted for 50% and beam walk accounted for 50% of the final score. The effect size (odds ratio) was calculated by a Chi square test using the Woolf logit interval for computing the 95% confidence interval, stratifying mice in terms of good outcome (defined as a score ≥ 3) versus bad outcome (score < 3). Odds ratios are reported in the forest plot and quantify the strength of the association between the genotype and the TBI outcome. Statistical analysis was performed with the standard software package GraphPad Prism (GraphPad Software Inc., San Diego, CA, USA, version 7.0); *p* values lower than 0.05 were considered significant.

### Experimental design and additional statistics

Mice were randomly allocated to surgery and assigned across cages and days. To minimize variability, all surgeries were performed by the same investigator. Subsequent behavioural, histological, immunohistological, and biochemical evaluations were performed blind by another investigator. Group size is of 14 defined by the formula: n = 2σ^2^f (α, β)/Δ^2^ (SD in groups = σ, type 1 error α = 0.02, type II error β = 0.2, percentage difference between groups Δ = 20). Standard deviation to be used in the formula for each assessment was calculated based on previous experiments with same outcome measures (e.g. behavioural deficits), resulting in σ = 16.9 and n = 14.4. To limit the use of animals, a post hoc power analysis test was done at n = 7 on raw data for each experimental branch. The experiment was interrupted at n = 7 for MBL-C^−/−^ and MBL-A^−/−^ mice since it was unable to provide significant differences using a reasonable number of animals (Δ = 3.89, σ = 22.49, thus expected n = 668.37). For MASP-2^−/−^ mice the experiment was interrupted at n = 7 since a significant difference was already in place due to a strong protective effect (Δ = 17.76 (3 week) Δ = 19.4 (4 week)).

Groups were compared by analysis of variance and post hoc testing as indicated in each figure legend. A parametric or nonparametric test was selected after the Kolmogorov–Smirnov test for normality to assess whether the data for the groups were normally distributed. The constancy of the variances was checked by the Bartlett test.

## Results

### MASP-2^−/−^ mice showed the best outcome after TBI with reduced sensorimotor deficits

**Fig. 1:**
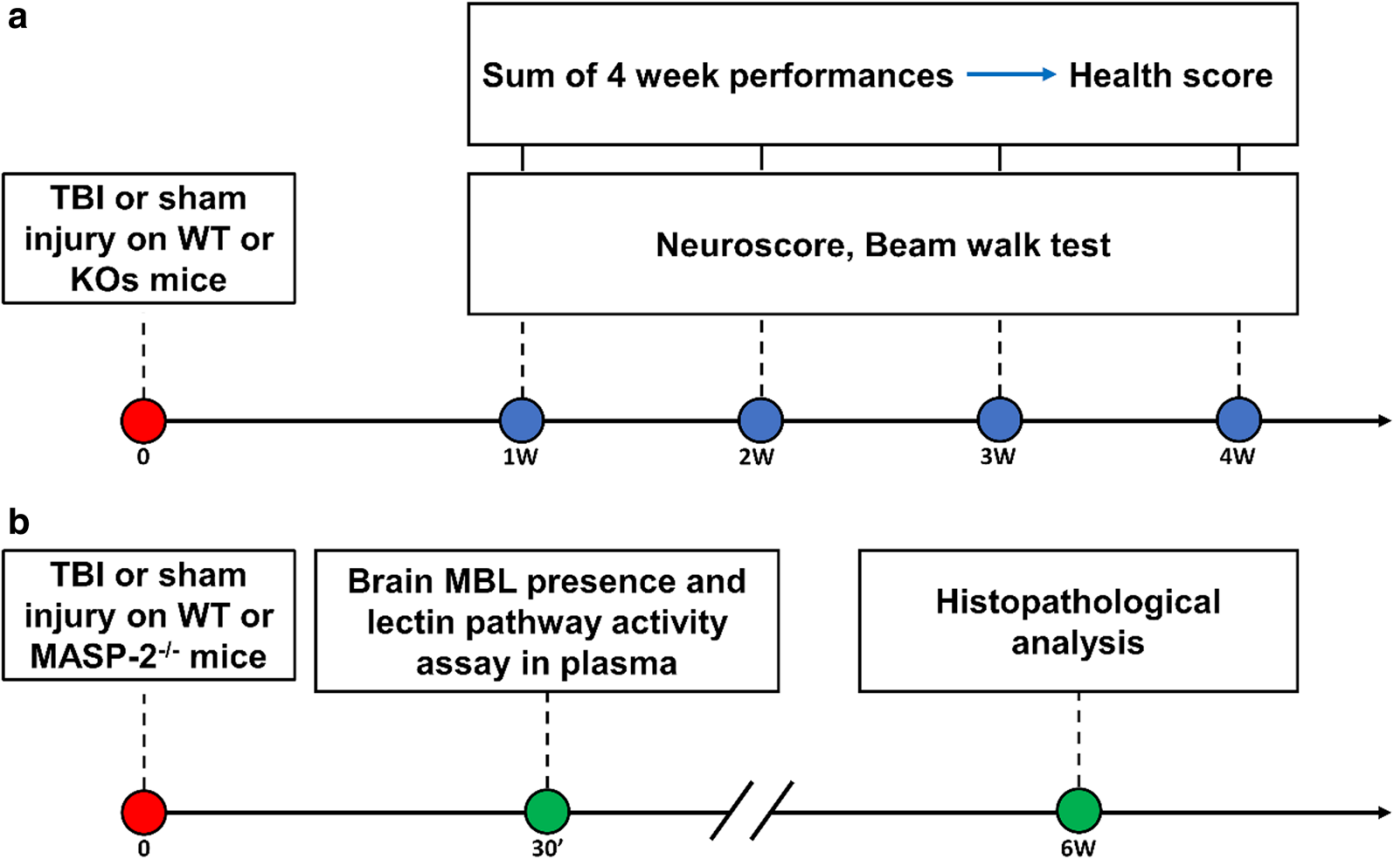
Experimental design. **a** WT or KOs mice (including: MASP-2^−/−^, MBL^−/−^, FCN-A^−/−^, CL-11^−/−^, MBL-C^−/−^, MASP-1/3^−/−^ and MBL-A^−/−^) were subjected to TBI or sham injury. Sensorimotor deficits were assessed weekly by neuroscore and beam walk test. The sum of 4-week performances of each mouse genotype has been used to calculate the health score. **b** MBL brain presence and residual LP activity in plasma was assed in MASP-2^−/−^ and WT TBI mice 30′ after surgery. Histopathological analysis was done for MASP-2^−/−^ and WT mice at 6 weeks after TBI

**Fig. 2:**
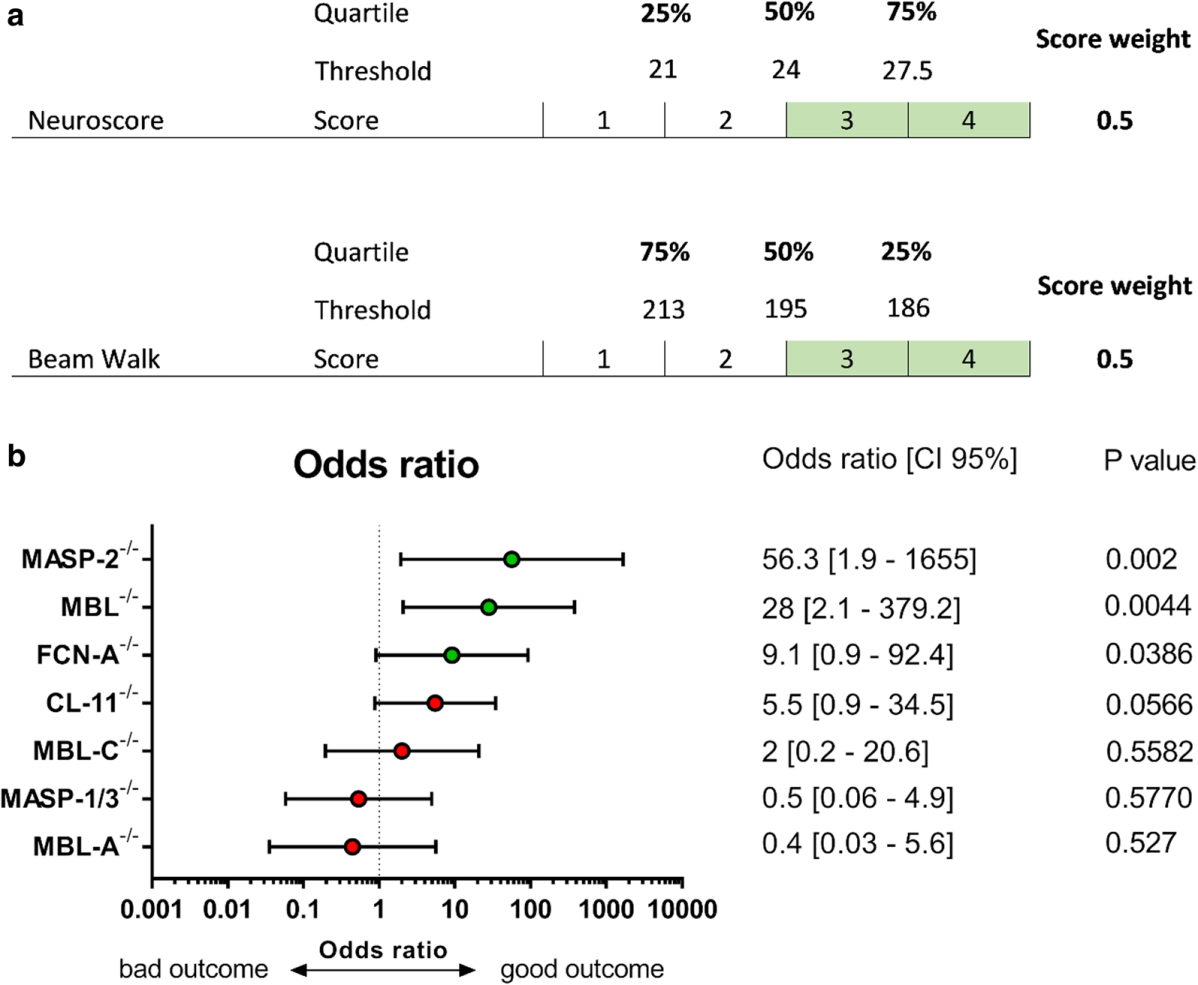
Association of LP protein deficiency with the behavioral outcome of traumatized mice over 4 weeks. **a** The health score was obtained by rating mice from 4 (good outcome) to 1 (bad outcome) based on quartiles of the neuroscore and beam walk values over the 4 weeks of observation. The final score was the sum of the weighted scores of the two parameters, e.g. the neuroscore and beam walk each accounted for 50% of the final score. **b** Association (odds ratio; 95% confidence interval) between the genotype and health score. The odds ratio was calculated by a Chi square test using the Woolf logit interval for calculating the 95% confidence interval (CI 95%), stratifying mice in terms of good outcome (defined as a score ≥ 3) versus bad outcome (score < 3). MASP-2^−/−^, MBL^−/−^ and FCN-A^−/−^ mice were significantly associated with good outcome scores

This study was conducted according to the plans depicted in Fig. 1a. Wild-type (WT) or KO mice (including: MASP-2^−/−^, FCN-A^−/−^, CL-11^−/−^, MBL-C^−/−^, MASP-1/3^−/−^ and MBL-A^−/−^) were subjected to TBI or sham injury. Sensorimotor deficits were assessed weekly for 4 weeks by neuroscore and beam walk tests. The data related to the sensorimotor performance are summarized in Table 1. The health score and odds ratio data for MBL^−/−^ mice were calculated using previously published data from our lab [32]. To obtain a comparative evaluation of the transgenic lines, a health score based on the outcome of the two sensorimotor tests was calculated. Mice were rated from 1 (bad outcome) to 4 (good outcome, Fig. 2a).

**Table 1:** Summary table

Strain	1 week		2 week		3 week		4 week	
	% of WT	*p* value	% of WT	*p* value	% of WT	*p* value	% of WT	*p* value
MASP-2^−/−^								
Neuroscore	+ 11.4	0.6079	+ 4.3	0.9605	+ 13.6	0.4541	− 5.9	0.8809
Beam Walk	+ 5.5	0.9608	+ 27.9	0.0681	+ 32.1	0.0202	+ 36	0.0091
MBL^−/−^								
Neuroscore	+ 33.7	0.0997	+ 42.8	0.0023	+ 37.2	0.0011	+ 35.6	0.0003
Beam Walk	+ 12.9	0.2972	+ 24.03	0.0131	+ 25.1	0.0133	+ 21.4	0.0742
FCN-A^−/−^								
Neuroscore	+ 19.7	0.9253	+ 0.37	> 0.9999	− 7.9	0.7784	+ 5.3	0.9171
Beam Walk	+ 6.7	0.9253	+ 15	0.5412	+ 13	0.685	+ 9.9	0.829
CL-11^−/−^								
Neuroscore	+ 16	0.4086	+ 13.5	0.5552	+ 11.2	0.631	+ 11.9	0.5552
Beam Walk	− 4.9	0.8735	+ 11.1	0.3198	+ 15.8	0.0711	+ 23.5	0.0024
MBL-C^−/−^								
Neuroscore	+ 10	> 0.9999	+ 2.9	> 0.9999	+ 11.1	> 0.9999	− 16.7	0.8796
Beam Walk	− 3.5	> 0.9999	− 3.5	> 0.9999	3.6	> 0.9999	+ 3.9	> 0.9999
MASP-1/3^−/−^								
Neuroscore	+ 9.1	0.9426	+ 11.9	0.784	+ 8.7	0.8759	− 1.9	0.9976
Beam Walk	+ 1.07	0.9997	+ 0.56	> 0.9999	− 2.6	0.997	0	> 0.9999
MBL-A^−/−^								
Neuroscore	+ 4.5	0.9986	+ 7.1	0.989	− 2.9	0.9986	− 19.5	0.589
Beam Walk	− 10	0.9025	− 4.2	0.9932	− 9.8	0.9133	− 11.01	0.8786

Summary of the outcome measures in the MASP-2^−/−^, MBL^−/−^, FCN-A^−/−^, CL-11^−/−^, MBL-C^−/−^, MASP-1/3^−/−^ or MBL-A^−/−^ TBI mice. For MBL^−/−^, data were obtained from the previous paper by Longhi et al. [32]

MASP-2^−/−^ was the most protective genotype, showing a positive association with a good outcome (odds ratio 56.3 [95% CI 1.9–1655], *p* = 0.002). MBL^−/−^ and FCN-A^−/−^ mice were also significantly protected, showing a positive association with a good outcome, but to a lesser extent than MASP-2^−/−^ (*p* = 0.0017 and *p* = 0.0386; odds ratio 36 [2.7–476.3] and 9.1 [0.9–92.4], respectively). CL-11^−/−^ and MBL-C^−/−^ genotypes showed a weaker, non-significant association with a good outcome (*p* = 0.0566 and *p* = 0.5582 respectively), whereas MASP-1/3^−/−^ and MBL-A^−/−^ mice were not protected (*p* = 0.5570 and *p* = 0.527 respectively, Fig. 2b).

### MASP-2^−/−^ mice had higher neuronal density than WT mice after TBI

**Fig. 3:**
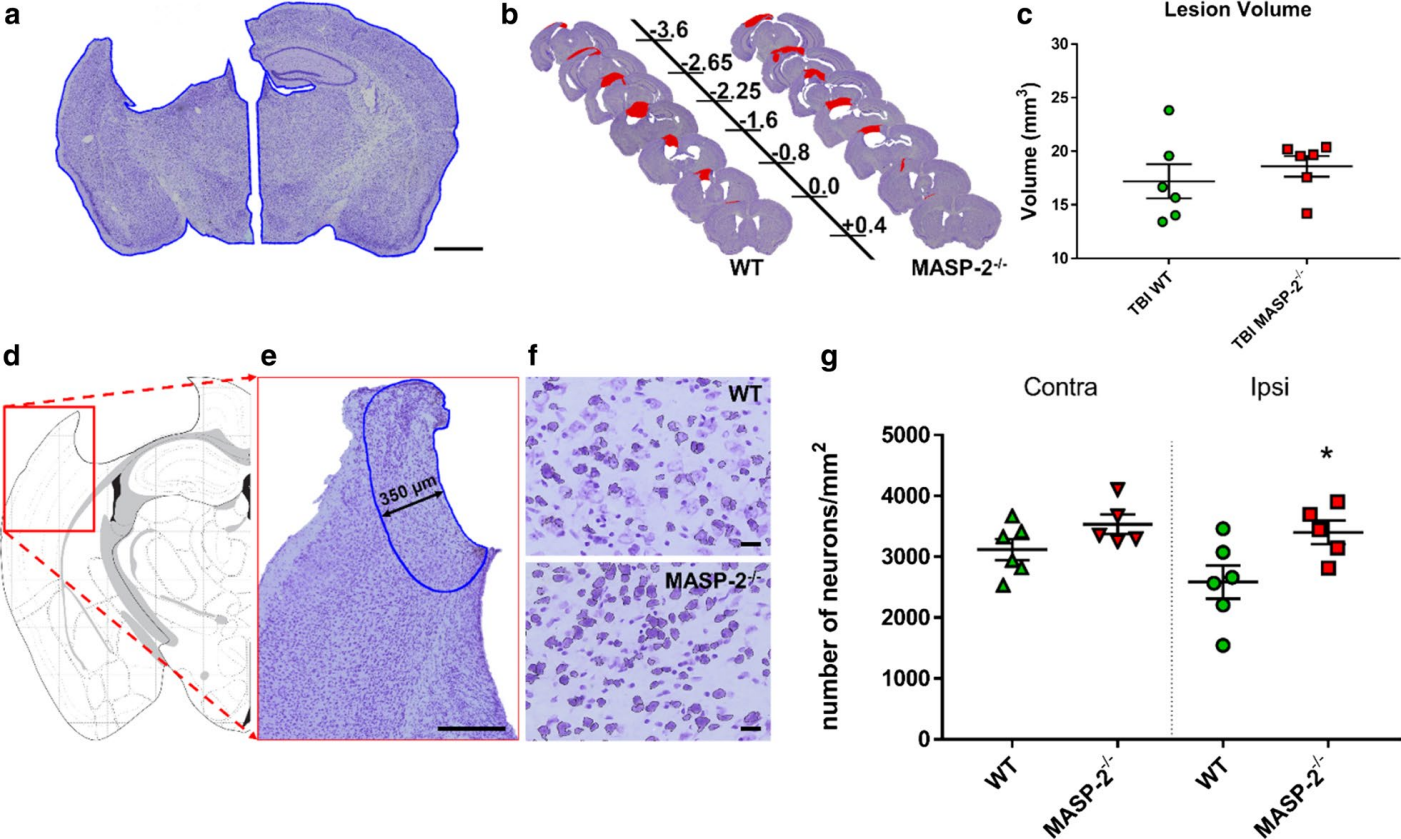
Histological analysis of lesion at 6 weeks after TBI in WT and MASP-2^−/−^ mice. **a** Representative image of Cresyl violet staining in ipsilateral and contralateral hemisphere. The lesion volume was evaluated based on the different extents of the ipsilateral and contralateral hemispheres, outlined in blue. Scale bar 1 mm. **b** Representative images of quantified sections for lesion volume at 6 weeks after TBI in WT and MASP-2^−/−^ mice. Distance from bregma in mm is indicated. **c** Quantification of the lesion showed no differences between WT and MASP-2^−/−^ mice 6 weeks after TBI. The data is shown as a scatter dot plot, line at mean ± SEM (*n* = 6); Mann–Whitney test. **d** Anatomical location of area of interest (red box). **e** Positioning of the cortical region of interest (blue outline) for calculating neuronal cell viability, traced at a distance of 350 μm from the contusion edge. Scale bar 350 μm. **f** 20 × high magnified fields of view showing a higher presence of neurons in MASP-2^−/−^ than WT mice. Scale bars 20 μm. **g** Quantification of neuronal density in the region of interest. MASP-2^−/−^ mice had higher neuronal density in the ipsilateral cortex than WT mice. The data is shown as a scatter dot plot, line at mean ± SEM (*n* = 5–6); Two-way Anova followed by Sidak’s post hoc test, *p < 0.05 compared with ipsi WT

Based on the outcome data, we focused on the MASP-2^−/−^ genotype and assessed the lesion size at 6 weeks (neuronal density, GFAP and CD11b immunostaining) and the degree of local brain inflammation at 30 min (presence of MBL-C in the brain and residual LP activity in plasma) (Fig. 1b). At 6 weeks post injury, we observed an extensive macroscopic area of cortical tissue loss, extending rostrocaudally from bregma + 0.4 to − 3.6 mm, both in WT and MASP-2^−/−^ injured mice, without differences between the two genotypes (17.2 ± 1.6 vs 18.6 ± 1.0 mm^3^ ± SEM Fig. 3a–c). We then assessed the neuronal density in a cortical region traced at a distance of 350 μm from the contusion edge (Fig. 3d, e) and in the corresponding contralateral hemisphere. MASP-2^−/−^ mice had higher neuronal density than WT mice at 6 weeks after TBI (31.5%, *p* < 0.05, Fig. 3g), indicating that the absence of MASP-2 functional activity was significantly protective against neuronal death after TBI.

### MASP-2^−/−^ mice did not show different astrogliosis and microglial activation after TBI

**Fig. 4:**
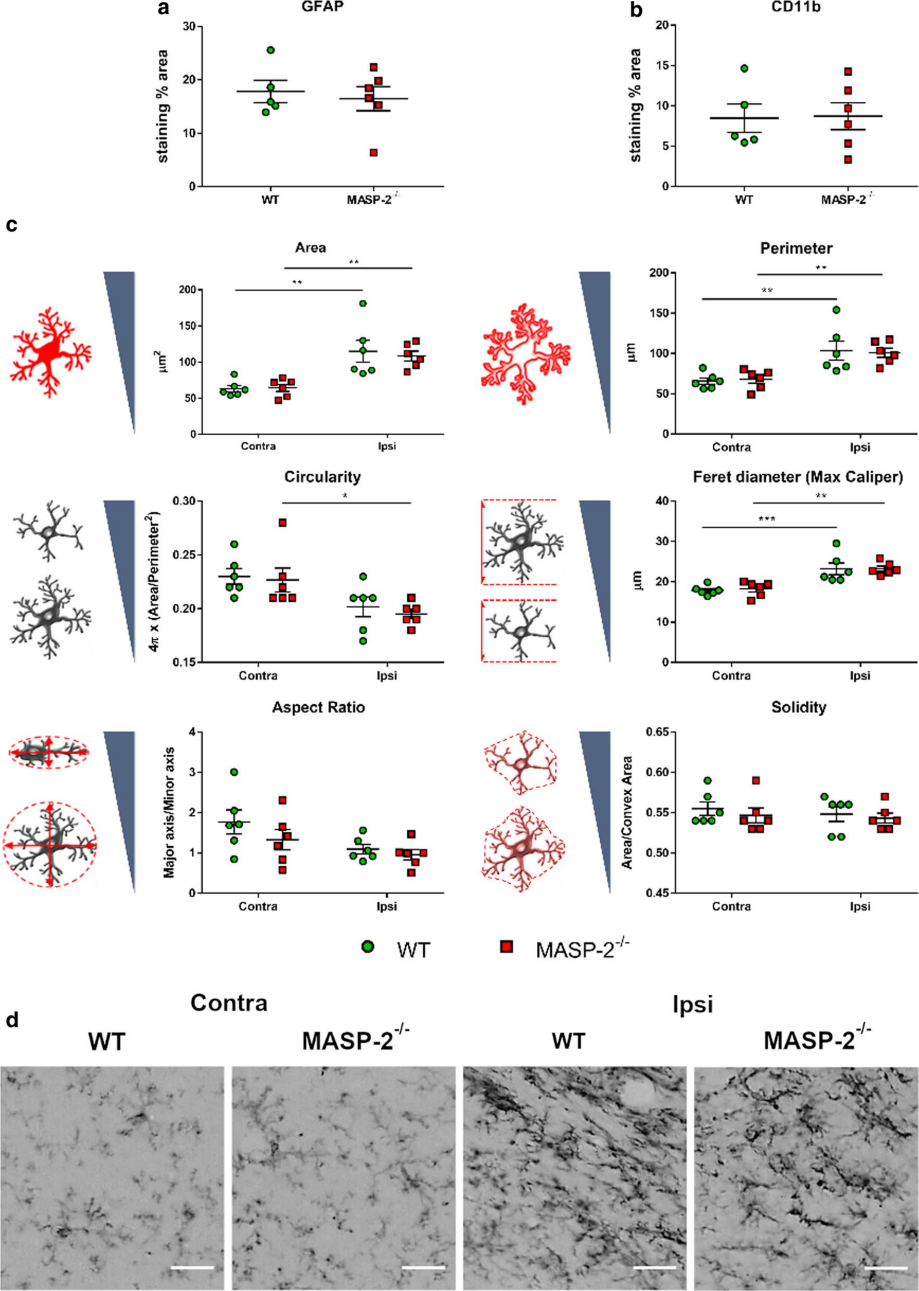
Inflammation markers and microglia shape descriptors 6 weeks after TBI in WT and MASP-2^−/−^ mice. **a**, **b** MASP-2 deletion did not affect the level of inflammatory markers such as glial fibrillary acidic protein (GFAP) and CD11b. The data is shown as a scatter dot plot, line at mean ± SEM (*n* = 5–6); unpaired *t* test. **c** Shape descriptors of CD11b positive cells showed that at 6 weeks after TBI microglia are still activated, having increased area, perimeter, Feret diameter and ramifications (indicated by lower circularity) higher in ipsi than in contralateral side. The drawings beside the y-axis indicate the expected values for each parameter depending on cell shape or symmetry. Microglia morphology did not differ between the two genotypes. The data is shown as a scatter dot plot, line at mean ± SEM (*n* = 6); Two-way Anova followed by Sidak’s post hoc test. **d** Representative high-magnification images of CD11b positive cells showing activated microglia in the ipsilateral side of both WT and MASP-2^−/−^ TBI mice, scale bar 20 µm

At 6 weeks after TBI, we measured astrogliosis and microglial activation quantifying the GFAP and CD11b immunopositive area at the edge of the contusion area. WT and MASP-2^−/−^ injured mice showed comparable astrogliosis (17.8 ± 2.1 vs 16.5 ± 2.3 staining  % area ± SEM) and microglial activation (8.5 ± 1.8 vs 8.7 ± 1.7 staining  % area ± SEM) when assessed as the total stained area (Fig. 4a, b). Since microglia morphology is indicative of the state of microglial activation, we subsequently analysed microglial cell shape descriptors [21]. Our analysis revealed that the proinflammatory morphology of microglia persists for as long as 6 weeks after TBI. Increased size (area, perimeter and Feret diameter) and higher ramified morphology (lower circularity) were observed in the ipsilateral region compared to the contralateral hemisphere (*p* < 0.01). However, no differences in microglial morphology were observed when comparing WT and MASP-2^−/−^ mice (Fig. 4c, d).

### MASP-2^−/−^ mice had impaired Lectin Pathway activation and normal MBL-C deposition

**Fig. 5:**
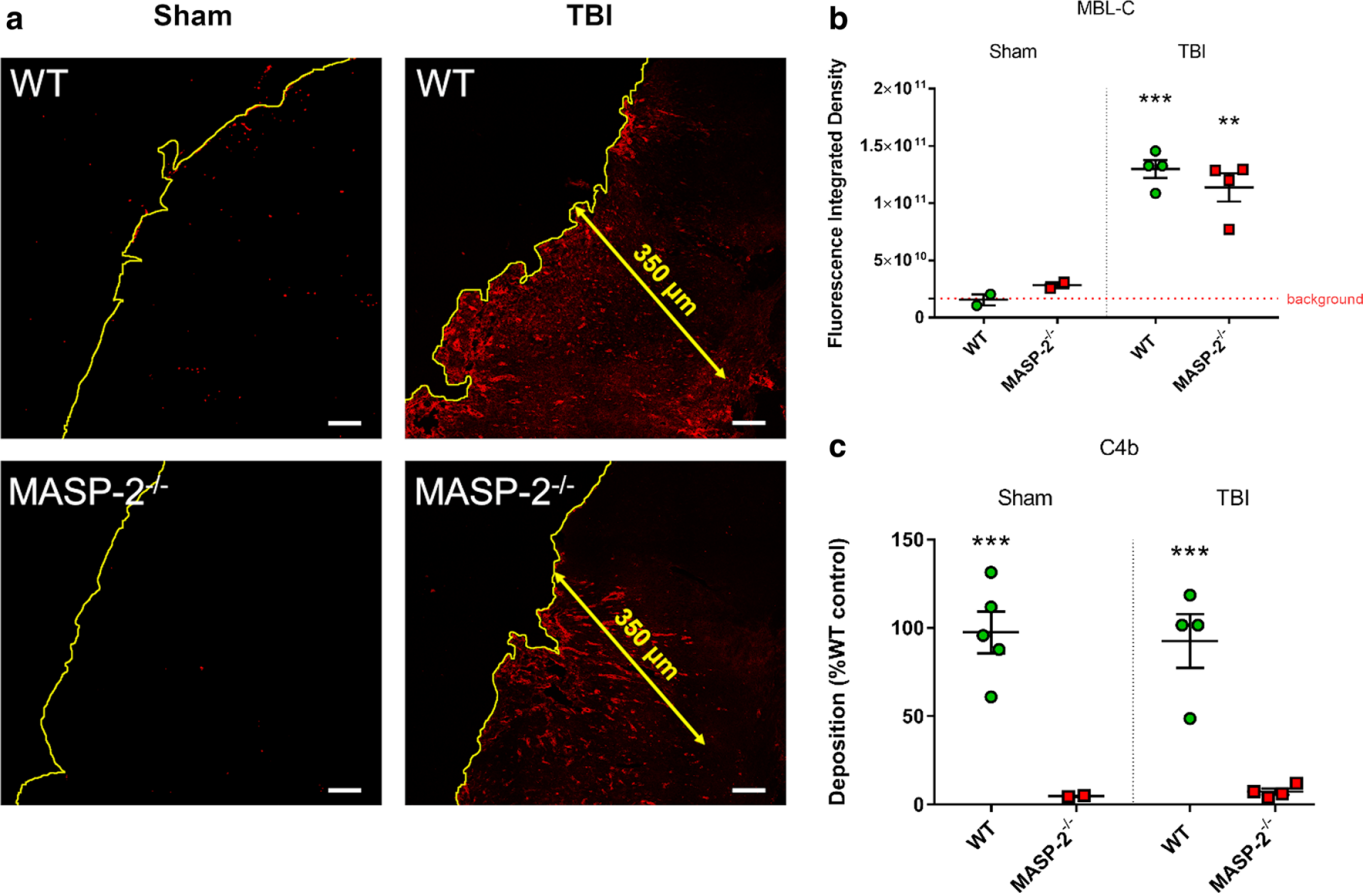
Brain MBL-C deposition and plasmatic LP activation 30′ after TBI in WT or MASP-2^−/−^ mice. **a** Representative low-magnification images of MBL-C immunolabeling at 30′ after TBI or sham surgery (the cortical edge is outlined in yellow). MBL-C quantification was done over an area of 350 µm from the contusion edge (Fig. 2). Scale bars 50 μm. **b** MBL-C deposition in brains of MASP-2^−/−^ mice was similar to that of WT. Data is shown as a scatter dot plot, line at mean ± SEM (*n* = 2–4). Two-way Anova followed by Sidak’s post hoc test, **p <0.01 compared with Sham MASP-2^−/−^, ***p < 0.001 compared with Sham WT. **c** In vitro assay for MBL-driven LP activation on mannan—plasma from MASP-2^−/−^ lack C4 convertase activity, resulting in minimal C4b deposition compared to WT mice. The data is shown as a scatter dot plot, line at mean ± SEM (*n* = 2–4), Two-way Anova followed by Sidak’s post hoc test, ***p <0.001 compared with Sham or TBI WT

Since MBL-C deposition can be detected in the brain after TBI [32], we measured the relative amount of MBL-C deposits in WT and MASP-2^−/−^ injured mice 30 min (′) after injury (Fig. 5a). We observed an increased deposition of MBL-C in TBI mice (compared to sham), regardless of the genotype (WT vs MASP-2^−/−^ 1.30 (± 0.08) × 10^11^ vs 1.10 (± 0.12) × 10^11^ fluorescence integrated density ± SEM Fig. 5b) indicating that the absence of MASP-2 did not affect the deposition of MBL-C. We then tested LP functional activity in mouse plasma using a functional in vitro assay [37] on mannan-coated plates, which measures LP activation through MBL (92.6 ± 15.2 vs 7.3 ± 1.7 optical density ± SEM) [38]. The absence of C4b deposition shows that MBL driven LP activation does not occur in MASP-2^−/−^ TBI mice, indicating that not even residual activation of the LP is occurring in MASP-2^−/−^ mice following TBI (Fig. 5c).

## Discussion

This study compares the long-term outcome after experimental TBI in mouse lines with targeted deficiencies of the LP-specific components, namely the recognition molecules ficolin-A, CL-11, MBL-C and MBL-A (both individually and combined) and the serine-proteases MASP-2, MASP-1 and MASP-3. To obtain a comparative outcome analysis, we developed a health score based on the neuroscore and beam walk test, two behavioural analyses evaluating sensorimotor deficits, over 4 weeks of observation. We found that MASP-2^−/−^, MBL^−/−^ and FCN-A^−/−^ mice had lower neurological deficits after TBI, indicating that these LP components are actively involved in driving the traumatic lesion and identifies them as possible pharmacological targets to reduce post-traumatic loss of functional activity and to improve recovery and clinical outcome.

MASP-2^−/−^ mice had the strongest reduction in sensorimotor deficits after TBI, which is in line with the key role of this enzyme in driving LP dependent complement activation. Of note, 6 weeks after injury, MASP-2^−/−^ TBI mice had higher neuronal density in the ipsilateral cortex compared to WT TBI mice, an important result given the severity of the TBI model used which has a major impact on neuronal viability and leads to major brain tissue loss. This is similar to what is observed in patients suffering from severe injury [23].

Our data underline the importance of the LP in TBI pathophysiology and identify MASP-2 as a key enzyme. Interestingly, neither of the other LP-associated enzymes, i.e. MASP-1 or MASP-3 appear to be involved in the posttraumatic loss of CNS functions following TBI, since MASP-1/3^−/−^ mice suffered from a similar degree of sensorimotor deficits than their WT controls. While it is accepted that only MASP-2 can generate the C3 convertase of the LP (C4bC2a) through its ability to cleave both C4 and C2 [39, 40], MASP-1 can support MASP-2 functional activity by cleaving C2 and enhance the conversion of zymogen MASP-2 into its enzymatically active form [41, 42].

It has been postulated that MASP-1 is an essential activator of MASP-2 [43] based on in vitro experiments using MASP-1/3 or MASP-1 deficient sera or sera treated with MASP-1 specific inhibitors. However, in in vivo models of MASP-2 dependent pathophysiology, such as ischemia reperfusion injury and TBI (reported here), the requirement for MASP-1 to activate MASP-2 appears to be negligible, since the absence of MASP-1 (or MASP-3) does not protect from MASP-2 dependent tissue injury. MASP-1 facilitates the conversion of zymogen MASP-2 into its enzymatically active form, most likely because the relatively low abundance of MASP-2 compared to MASP-1 limits LP-specific trans-activation events [44, 45]. In an experimental model of ischemic stroke, we reported that combined deficiency of MASP-1 and MASP-3 did not affect the ischemic outcome, while that of MASP-2 was protective [18]. Therefore, we hypothesized that ischemic brain injury involved a MASP-2 dependent pathophysiological process, thus underlining that MASP-1 is not an essential activator of MASP-2. Consistent with this conclusion is our present observation that the total absence of LP-mediated complement activation in MASP-2^−/−^ mice is protecting from post-traumatic injury, while MASP-1/3 double deficient mice show no degree of protection. In this context, it is important to keep in mind that MASP-2 can also cleave C3 directly through a C4 and C2 independent bypass activation of native C3 [46].

Our study shows that MBL^−/−^ and FCN-A^−/−^ mice were significantly associated with a good outcome score indicating that they may be the relevant initiator molecules. In line with this, we have previously shown the effectiveness of targeting MBL in TBI mice by inhibiting MBL with Polyman9, a polymannosylated compound, which binds to the carbohydrate recognition domain of MBL, attenuating sensorimotor deficits up to 4 weeks after TBI [28, 32]. As for ficolin-A, the mouse orthologue of human ficolin‐2, no data are available on its role in the context of brain injury. In TBI patients ficolin-2 has been found in the peri-contusional brain area at significantly higher levels than in the brain from control patients [23]. Its role in TBI has to be further investigated.

A common feature of the initiators of the lectin complement pathway is their ability to interact with MASP-2 to trigger the activation of the LP. Thus, MASP-2 should be a more effective target than one of the five LP initiators. The data obtained in this work supports this notion, as MASP-2 deficiency provides better protection against TBI damage than that of a specific initiator. However, as previously reported in models of ischemia/reperfusion injury, recognition molecules can have detrimental effects independently from the LP cascade activation [16, 47]. In particular, MBL itself has been shown to promote vascular injury on endothelial cells having undergone hypoxic stimulus by direct action—including structural damage to the endothelial cell cytoskeleton—without requiring LP activation [16]. Consistent with this finding, genetic deletion of MBL or its pharmacological inhibition were sufficient to obtain significant protection in experimental models of ischemic stroke, where vascular dysfunction is critical [17, 20, 48]. The fact that in experimental TBI, MBL depletion provided a lower degree of protection than MASP-2 depletion may suggest that the complement-independent vascular effects of MBL are less important for TBI pathophysiology. In line with this suggestion, the presence of MBL within the TBI-injured area was not different in WT and MASP-2^−/−^ mice, supporting the hypothesis that LP-independent detrimental effects of MBL have a limited contribution to TBI sequelae. Moreover, most of the MBL in the lesion core area was extravascular, implying that MBL-driven vascular effects may be marginal, at variance with what happens in ischemic injury.

Microglia activation may contribute to specific behavioural deficits, like sickness behaviour in mice, corresponding to depressive behaviours seen in TBI patients, months after injury [49, 50]. Here we report that 6 weeks after TBI, microglia are still activated, showing increased size and ramifications in ipsi- compared to contra-lateral side, with no difference between WT and MASP-2^−/−^ mice. We previously found that early (48 h) after ischemia/reperfusion injury MASP-2 deletion was associated with microglia presenting typical anti-inflammatory morphological features, as opposed to the hypertrophic microglial morphology associated with the phagocytic activity and pro-inflammatory state present in ischemic WT brains [18]. We cannot exclude that MASP-2 deletion affected microglia activation at earlier time points after TBI, with consequences on the overall outcome. However, data show that this effect is no more detectable at chronic time points.

It is known that the complement system drives microglia-driven synaptic control in memory loss [51] and in chronic neurodegenerative disease like Alzheimer’s [52], a possible long-term consequence of TBI [53]. However complement-mediated synapse elimination appears to be associated with proteins of the classical pathway, particularly C1q, released locally in the brain [52, 54], with no specific involvement of the LP, as shown here.

The results presented here, obtained in experimental TBI, pointing to MASP-2 as a key enzyme in TBI pathology, are in line with what is observed in TBI patients. Namely, we previously reported that: (1) the complement system is fully activated down to the level of the formation of the terminal complement complex; (2) the lectin pathway components are persistently present, up to 5 days post-TBI; and (3) MASP-2 in the brain is significantly increased and associated with TBI severity, indicated by abnormal pupil reactivity and traumatic subarachnoid haemorrhage [23]. Moreover others reported that increased circulating levels of MASP-2 are associated with a poor outcome at 90 days after injury [55].

It should not be excluded that other pathways of complement system may have a role in propagating chronic post-TBI pathology. Indeed, we also reported an increased presence of MASP-3 in the brains of TBI patients compared to control patients [23], supporting a role of the alternative pathway in TBI. Indeed pharmacological inhibition of the alternative pathway provided significant improvements in histological, and functional recovery in an experimental model of TBI [56].

## Conclusion

This study highlights the significant contribution of LP to the post-traumatic inflammatory pathology following TBI and shows that the highest degree of protection is achieved through the absence of the LP key enzyme MASP-2, underlying the therapeutic utility of MASP-2 targeting following acute brain injury. This points to LP and MASP-2 as novel pharmacological targets for TBI, thus paving the way for future development of clinical strategies. MASP-2 represents a unique target: it is a single, low-abundance enzyme that is exclusively synthesized in the liver [57], so the effectiveness of systemic MASP-2 inhibitory agents is not complicated by local biosynthesis in the brain and the limitations imposed by the blood–brain barrier. Of note, an inhibitory antibody against MASP-2 (Narsoplimab) is currently undergoing phase 3 clinical trials for the treatment of Hematopoietic Stem Cell Transplant-Associated TMA (ClinicalTrials.gov Identifier: NCT04247906), IgA Nephropathy (NCT03608033) and Atypical Hemolytic Uremic Syndrome (NCT03205995) and in a phase 2 clinical trial for the treatment of Lupus Nephritis (NCT02682407), thus making the present work potentially transferable to the clinical setting in the near future.

## Data Availability

The data sets generated and/or analyzed during the current study are available in the Figshare repository, 10.6084/m9.figshare.12416597.

## Abbreviations

LP: Lectin pathway
WT: Wild-type
TBI: Traumatic brain injury
BBB: Blood–brain barrier
DAMPs: Damage-associated molecular patterns
